# Core Outcome Set and Reporting Checklist for Studies on Vasa Previa

**DOI:** 10.1001/jamanetworkopen.2025.1000

**Published:** 2025-03-18

**Authors:** Tiffany Yeretsian, Nasrin Javid, Natasha Hirschhorn-Edwards, Rizwana Ashraf, Alisha Adams, John Kingdom, Rohan D’Souza

**Affiliations:** 1 Department of Obstetrics and Gynecology, Mount Sinai Hospital, University of Toronto, Toronto, Ontario, Canada; 2 Department of Obstetrics and Gynecology, McMaster University, Hamilton, Ontario, Canada; 3 Royal College of Surgeons in Ireland, Dublin, Ireland; 4 Sydney Institute for Women, Children and Their Families, Sydney Local Health District, Sydney, New South Wales, Australia; 5 South Western Sydney Local Health District, Liverpool, New South Wales, Australia; 6 Faculty of Health, University of Technology Sydney, Sydney, New South Wales, Australia; 7 School of Population Health, University of New South Wales, Sydney, Australia; 8 International Vasa Previa Foundation, Chester, Illinois; 9 Department of Health Research Methods, Evidence and Impact, McMaster University, Hamilton, Ontario, Canada

## Abstract

**Importance:**

Vasa previa, although a rare condition affecting 0.46 per 1000 pregnancies, results in high rates of fetal and neonatal mortality and morbidity. Widespread use of a core outcome set and reporting checklist may enable data harmonization, enhance evidence synthesis, and ensure prioritization of patient-important outcomes in clinical practice, future research, and clinical practice guidelines.

**Objective:**

To develop a core outcome set for studies on vasa previa that includes perinatal screening, diagnosis, and management of vasa previa.

**Design, Setting, and Participants:**

This survey study involved a 2-round international and cross-disciplinary Delphi survey and virtual consensus meetings conducted between March 14, 2021, and October 4, 2022. Health care professionals (HCPs) and health service users (HSUs) were invited to participate in the development of the core outcome set.

**Exposure:**

Perinatal screening, diagnosis, and management of vasa previa.

**Main Outcome and Measures:**

Core outcomes and reporting checklist items for future studies on vasa previa were developed using Delphi surveys, small group discussions, and a consensus meeting.

**Results:**

An international group of 204 participants, comprising 115 HSUs and 89 HCPs representing 27 countries, rated 67 outcomes identified through a systematic review and qualitative interviews in a 2-round online Delphi survey. Through virtual small group discussions and a consensus meeting, 13 core outcomes were identified: pregnancy outcome (live birth, fetal loss [miscarriage or stillbirth], or neonatal death), severe neonatal morbidity, fetal/neonatal blood loss, neonatal intensive care unit admission for >24 hours, gestational age at birth, third trimester confirmation of vasa previa diagnosis, neurocognitive and developmental outcomes, mode of birth, maternal death or severe morbidity, maternal quality of life, duration of antepartum admission, and antepartum or peripartum hemorrhage. A 22-item reporting checklist was simultaneously developed to include important items in studies on vasa previa when possible and/or relevant.

**Conclusions and Relevance:**

The core outcome set and reporting checklist for vasa previa are hoped to encourage researchers to incorporate patient-important outcomes in future studies to enable data harmonization, improve evidence synthesis, and facilitate the development of clinical practice guidelines for this rare and highly lethal condition for which timely diagnosis and management may prevent fetal and maternal morbidity and mortality.

## Key Points

**Question:**

What patient-important outcomes should be included in research studies on vasa previa, a rare and highly lethal condition for which timely diagnosis and management may prevent serious adverse outcomes?

**Findings:**

This survey study used a 2-round international Delphi survey, small group discussions, and a consensus meeting that involved 115 people with lived experience of vasa previa and 89 health care professionals from 27 countries. The participants identified 13 core outcomes and 22 reporting checklist items that researchers are encouraged to measure and report.

**Meaning:**

These findings suggest that there is global consensus between people with lived experience of vasa previa and health care professionals on core outcomes and other important data elements that should be reported in future studies on vasa previa.

## Teaser

This survey study used Delphi surveys, small group discussions, and consensus meetings to develop patient-important outcomes for future studies on perinatal screening, diagnosis, and management of vasa previa.

## Introduction

Vasa previa is a rare condition in which fetal blood vessels run outside the umbilical cord, within the placenta, and through the fetal membranes, crossing over or in proximity to the internal cervical opening.^1^ The positioning of fetal vessels places them at high risk of compression in late pregnancy, leading to abnormal fetal heart rate patterns and rupture of the vessels in the event of prelabor rupture of membranes, thereby increasing the risk of stillbirth, neonatal mortality, and serious fetal and neonatal morbidity. Although vasa previa is rare, affecting 0.46 per 1000 pregnancies (95% CI, 0.33-0.59 per 1000 pregnancies),^2^ it is estimated that 40% to 60% of newborns will not survive if vasa previa is not diagnosed prior to labor and vaginal birth.^2,3,4^ Early screening could lead to better management plans, but there is a lack of high-quality evidence to guide protocols for diagnosis and treatment due to heterogeneous outcome reporting in research studies.^5,6,7^ Furthermore, published studies have not adequately reported on patient-important outcomes.^8^ This underreporting has resulted in inconsistent diagnostic and treatment approaches among pregnancy care practitioners worldwide that are not patient-centered and contribute to maternal isolation, anxiety, and fear.^9,10,11^ One solution is to develop a core outcome set, a minimum set of outcomes considered important by families with lived experience of vasa previa (referred to as health service users [HSUs]) and health care professionals (HCPs) that researchers are encouraged to report in future studies while not preventing them from reporting other relevant outcomes.^12^ This study aimed to establish consensus between HSUs and HCPs in developing the Core Outcome Set for Studies on Vasa Previa (COVasP) to promote harmonized reporting of patient-important outcomes in research studies on vasa previa and provide more reliable data for future meta-analyses.

## Methods

This survey study involving Delphi surveys, small group discussions, and a consensus meeting and consent forms for stakeholder participation in the Delphi process were approved by the institutional research ethics boards at Mount Sinai Hospital and the University of Technology Sydney. Written informed consent was obtained from participants prior to participation in the Delphi survey and the small group discussions. This study adheres to the American Association for Public Opinion Research (AAPOR) reporting guideline for survey studies, including transparency in survey methodology, participant recruitment processes, and response rates. Specifically, we ensured clear communication of survey purposes, confidential data handling, and detailed reporting of sample recruitment strategies, participant characteristics, and nonresponse rates.^13^

The COVasP study is part of the Outcome Reporting in Obstetric Studies Project, an international research group comprising members from diverse disciplines, perspectives, and expertise focused on developing core outcome sets.^14^ A study protocol that followed a multistep process guided by the Core Outcome Measures in Effectiveness Trials (COMET) handbook^12^ and the Core Outcome Set–Standards for Development recommendations^15^ has been published separately.^16^ The COVasP study was prospectively registered on the COMET website.^17^

### Identification of Relevant Outcomes

Relevant outcomes were identified through 2 main sources: (1) a systematic review and (2) a qualitative study. The systematic review identified 74 outcomes from 160 studies involving 1004 pregnancies.^8^ The qualitative study involved interviews with 18 HSUs who had experienced vasa previa and 6 HCPs, resulting in the identification of 53 patient-important outcomes and experience measures.^18^ The COVasP study team reviewed and assessed the outcomes identified through these steps, removed duplicates, and grouped related outcomes through an iterative process. Ultimately, 67 unique outcomes were identified and informed the subsequent steps of core outcome set development: a 2-round online Delphi survey, small group discussions, and a final consensus meeting.

## Data Analysis

### The Delphi Survey

#### Participant Identification and Recruitment

Because of the rare nature of vasa previa and to capture the opinions of international stakeholders, participants for the Delphi survey were recruited between March 14 and April 28, 2021, through social media advertisements and relevant organizations specified in the study protocol. These organizations cover areas related to pregnancy, childbirth, maternal and infant health, obstetrics, gynecology, and vasa previa, including the International Vasa Previa Foundation.^16^ Additionally, corresponding authors of the 160 publications included in the systematic review were contacted between March and April 2021. Participants were categorized into 2 groups: (1) HSUs, including individuals with lived experience of vasa previa, family members, and patient advocates or representatives, and (2) HCPs, including clinicians, academics (eg, researchers, epidemiologists and methodologists, and core outcome set developers), and other stakeholders directly or indirectly involved in the care of pregnant individuals, such as hospital administrators, guideline developers, journal editors, and policy makers.

#### Delphi Survey Process

The survey was launched online on March 14, 2021, using DelphiManager software (COMET Initiative), an online platform ensuring privacy, feasibility, cost-effectiveness, and reliability while facilitating global representation.^12^ The survey link was disseminated through channels that included the International Vasa Previa Foundation mailing list, social media, and personalized emails to HCPs interested in vasa previa or high-risk obstetrics. Participants were given 6 weeks to complete the first round, with weekly reminder emails for those who registered but had not completed the survey.

Participants were first presented with a demographic questionnaire that included details on clinical specialty (if applicable), country of residence, and countries and clinical settings of practice or birth. Data on age, sex and gender, and race and ethnicity were not collected. The 67 unique outcomes identified through earlier steps were categorized into 5 core areas, including mortality, morbidity (clinical and physiologic outcomes), life impact (functioning), resource use, and adverse events, based on a taxonomy developed for outcomes in medical research.^19^ Lay explanations were provided for clinical terms to ensure clarity for nonclinicians. Participants rated each outcome on a 9-point Likert scale based on perceived importance: 1 to 3 points (not essential), 4 to 6 points (important but not critical), and 7 to 9 points (critically important for inclusion), with an option to select unable to score.^12^ Participants could also suggest additional outcomes not included in the provided list. New suggestions were reviewed by the research team and included in the second survey if deemed unique. Histograms of Likert scores from the first round of the Delphi survey, stratified by participant groups, were shared during the second round. Only participants completing the first round were invited to the second round. The second round commenced on May 15, 2021, and remained open for 6 weeks. Following the second Delphi survey, outcomes were classified as consensus in (>70% scoring 7-9 points), consensus out (>70% scoring 1-3 points), or no consensus.

### Small Group Discussions and Final Consensus Meeting

The COVasP study team reviewed the results of the 2-round Delphi survey and addressed the large number of outcomes for which consensus had not been reached. The team decided to amend the protocol and schedule virtual small group discussions prior to the final consensus meeting that involved HSUs and HCPs who had completed both Delphi rounds and responded to our invitation. Prior to the small group discussions, which were held in June 2022, participants were sent a list of items for which consensus was not reached and were asked to independently assess whether these items should be included as core outcomes or reporting checklist items or excluded. During the small group discussions, participants discussed their perspectives, deliberated, and collectively decided on the status of each outcome. Consensus from the Delphi survey, small group discussions, and final consensus meeting were recorded and tracked using R, version 4.3.0 (The R Foundation) and Excel for Macintosh, version 16.88 (Microsoft Corporation).

Due to global travel restrictions during the COVID-19 pandemic, the final in-person meeting was replaced with a virtual meeting held on October 4, 2022. Participants from the small group discussions and others interested in the final consensus process were invited. A summary of outcomes previously classified as consensus in or consensus out was presented before discussing outcomes still lacking consensus. All participants voted yes or no for each item to determine inclusion in the core outcome set. In line with established practices for core outcome set development, which suggest a lower threshold of 50% to 60% in later rounds to facilitate consensus while considering expert opinions,^12,20^ an item was included if 50% or more of the group voted yes. Members of the COVasP study team attended as observers, with T.Y. and R.D. serving as chairs.

## Results

**Figure 1.**
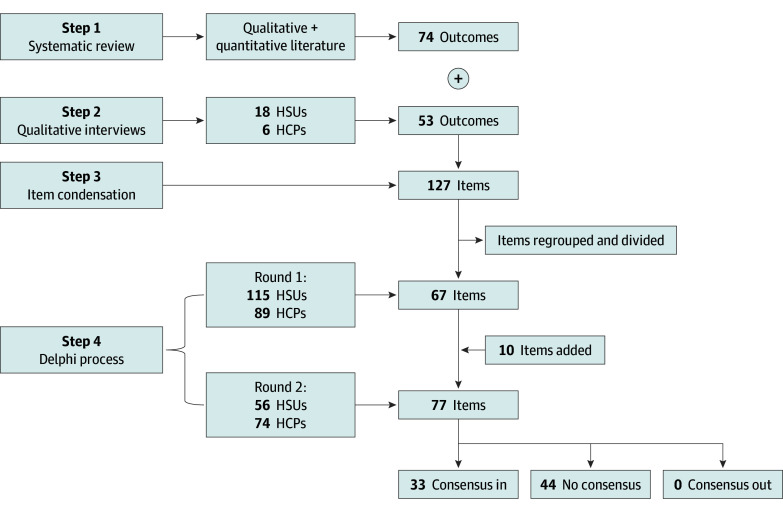
Core Outcome Set for Studies on Vasa Previa Outcome Decision Process: Systematic Review, Interviews, and Item Condensation HCP indicates health care professional; HSU, health service user.

The first round of the Delphi survey was completed by 204 participants from 27 countries, comprising 89 HCPs and 115 HSUs. Participant characteristics are detailed in Table 1. In addition to rating outcomes on a 9-point Likert scale, participants suggested 48 new outcomes. These outcomes were discussed by the COVasP study team, and after removing duplicates and merging similar outcomes, 10 items were added to the second round (Figure 1). The second round involved 130 participants (with a 36% attrition rate), including 74 HCPs and 56 HSUs. Of the 74 participants lost to follow-up, 15 initiated, but did not complete the second round despite multiple reminder emails, while 56 failed to engage with the second round entirely.

**Table 1.** Round 1 Delphi Survey Demographics

Characteristic	Participants, No. (%)
**Health care professionals (n = 89)**	
Specialty	
Obstetrics and gynecology	31 (34.8)
Maternal-fetal medicine	24 (27.0)
Midwifery	24 (27.0)
Academic/research	3 (3.4)
Other^a^	7 (7.9)
Years of clinical or academic experience	
0-9	16 (18.0)
10-19	22 (24.7)
20-29	33 (37.1)
≥30	15 (16.9)
Not disclosed	3 (3.4)
Continents practiced in	
Africa	4 (4.5)
Asia	8 (9.0)
Australia and Oceania	41 (46.1)
Europe	18 (20.2)
North America	20 (22.5)
South America	3 (3.4)
Not disclosed	1 (1.1)
No. of countries practiced in	
1	83 (93.3)
≥2	6 (6.7)
Location of practice	
Academic center	48 (53.9)
Community setting	
Academic urban	8 (9.0)
Nonacademic urban	29 (32.6)
Rural/country	3 (3.4)
Not disclosed	1 (1.1)
**Health service users (n = 115)**	
Experience with vasa previa	
Patients^b^	110 (95.7)
Patient advocate or family member	5 (4.3)
Highest level of education	
High school	17 (14.8)
College or university	51 (44.3)
Postgraduate education	41 (35.7)
Not disclosed	6 (5.2)
Continent where pregnancy care was received	
Australia and Oceania	16 (13.9)
Europe	22 (19.1)
North America	73 (63.5)
Not disclosed	4 (3.5)

^a^ Included anesthesiology, family practice, internal medicine, pediatrics, lactation consultation, and ultrasonography.

^b^ Six patients also identified as health care professionals but did not disclose their specialties. They completed the survey as health service users and not as health care professionals.

Following 2 rounds of the Delphi survey, 33 outcomes achieved consensus in (a score of 7-9 points on the Likert scale), while 44 outcomes lacked consensus (a score of 4-6 points). No items were rated as consensus out (a score of 1-3 points). The COVasP study team convened to decrease the number of items by merging similar items (eg, miscarriage and stillbirth were both consensus in and were merged into a single outcome, fetal loss) and reformulate wording for clarity (eg, length of hospital stay in the neonatal intensive care unit [NICU] was reformulated to “admission to the NICU or special care nursery for >24 hours [if yes, length of stay]”). On further discussion, the team determined that certain items were important to report in studies but did not adhere to the definition of outcomes in clinical research, which is “a measurement or observation used to capture and assess the effect of treatment such as assessment of side effects (risk) or effectiveness (benefits).”^12^^(p1)^ For example, risk factors for vasa previa, including placenta previa, low-lying placenta, succenturiate placental lobe, twins or higher-order pregnancies, and use of assisted reproductive techniques, are important to report, but they are not outcomes in the context of studies on vasa previa. These items were designated as reporting checklist items, which the COVasP study team defined as important items to report in studies on vasa previa where possible or relevant.

Through iterative refinement, the number of survey items was reduced to 36, and these were discussed during the small group discussions. Eight HSUs and 11 HCPs participated in 5 virtual small group discussions, representing diverse backgrounds from 7 countries, including Australia, Canada, Denmark, Ireland, Nigeria, the UK, and the US. These discussions enabled participants to debate and decide on each outcome’s inclusion. Following are 3 key discussions.

### Reassignment of Some Outcomes Into Reporting Checklist Items

Participants identified that in addition to the aforementioned risk factors, several other items, although important and readily available, were not directly related to vasa previa and should, therefore, be listed as reporting checklist items. Examples include whether antenatal corticosteroids were administered and the type of anesthesia or analgesia for birth.

### Relevance of Some Outcomes

Participants acknowledged that the relevance of some outcomes depended on whether antenatal screening had been performed. For example, ongoing antenatal screening for fetal anemia or cervical shortening are only relevant when antenatal screening is performed, whereas mode of birth (vaginal vs cesarean delivery) is only relevant in settings without routine screening, when vasa previa is diagnosed intrapartum or postpartum.

### Rewording Outcomes to Improve Generalizability

Participants stated that while it was important to confirm the antenatal diagnosis of vasa previa on histopathology, many clinical units did not have this facility. Besides, sometimes despite visualizing vasa previa intrapartum, histopathology reports did not agree. Therefore, it was deemed restrictive to depend on histopathology alone for confirmation of diagnosis. To improve generalizability, the outcome on confirmation of diagnosis was reworded to “confirmation of diagnosis via third trimester ultrasonography, direct visualization at the time of childbirth, or by histopathology.”

**Figure 2.**
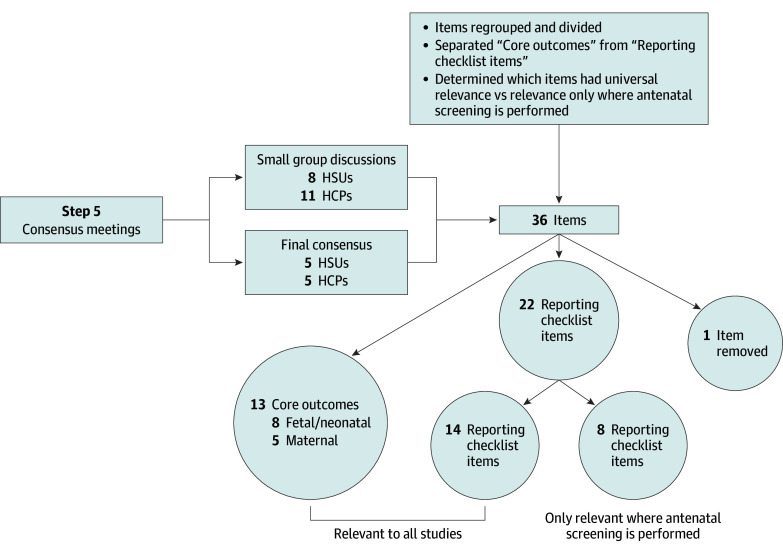
Core Outcome Set for Studies on Vasa Previa Outcome Decision Process: Delphi Consensus and Finalization HCP indicates health care professional; HSU, health service user.

After compiling results from the 5 small group discussions, consensus had been attained on all but 6 outcomes. These 6 were brought forward and discussed at the final consensus meeting. At this meeting, which included 5 HSUs and 5 HCPs, consensus was reached to include 13 items as core outcomes, including 8 fetal/neonatal (pregnancy outcome [live birth, fetal loss (miscarriage or stillbirth), or neonatal death], severe neonatal morbidity, fetal or neonatal blood loss, NICU admission for >24 hours, gestational age at birth, third trimester confirmation of vasa previa diagnosis, neurocognitive and developmental outcomes, and rupture of membranes) and 5 maternal outcomes (maternal death or severe morbidity, maternal quality of life, duration of antepartum admission, mode of birth, and antepartum or peripartum hemorrhage) (Table 2). Consensus was also reached on 22 items for the reporting checklist to be recorded in all studies where relevant (Table 3). These 2 checklists collectively represent the final output of the COVasP study and are available in fillable format (eAppendix in Supplement 1). A summary of the process and personnel involved at each step is presented in Figure 1 and Figure 2.

**Table 2.** Core Outcome Set for Studies on Vasa Previa

Core outcome	Explanations and reporting recommendations
Pregnancy outcome	Live birth, fetal loss (miscarriage or stillbirth), neonatal death
Severe neonatal morbidity	Specify definitions, indicators, conditions included
Fetal or neonatal blood loss	Blood loss from damage to or rupture of the fetal vessel resulting in exsanguination, anemia, hypovolemic shock, and/or need for blood transfusion
Gestational age at birth	Presented in weeks
Rupture of membranes	Yes/no; if yes, time interval between rupture of membranes and childbirth
Admission to the neonatal intensive care unit or special care nursery for >24 h	Yes/no; if yes, length of stay in d
Neurocognitive and developmental outcomes	Baby’s physical, neurodevelopmental, cognitive, and psychologic development after birth requires ongoing health care monitoring and testing
Third trimester confirmation of diagnosis of vasa previa	Confirmation of diagnosis is suggested through 1 of 3 approaches: (1) third trimester ultrasonogram, (2) direct visualization at the time of childbirth, or (3) histopathology of placenta and membranes
Maternal death (all cause) or severe maternal morbidity	For maternal mortality, specify time and cause of death; for severe maternal morbidity, specify definition and indicators used
Mother’s quality of life and perceived health status	Include instrument used, as well as timing and frequency of measurement
Duration of maternal antenatal hospitalization (in d)	Specify reasons for hospitalization
Mode of birth	Planned vs emergency cesarean delivery (mention indication if emergency cesarean delivery); vaginal delivery (mention indication [eg, resolution of vasa previa])
Antepartum or intrapartum hemorrhage	Yes/no; if yes, time interval between onset of vaginal bleeding and childbirth

**Table 3.** Reporting Checklist Items

Reporting checklist item^a^	Explanation and reporting recommendations
**Relevant to all studies on vasa previa**	
Availability and accessibility to treatment/interventions	Yes/no
Type of vessel (artery or vein) ruptured or in proximity to the internal cervical os	Yes/no; if no, why (ie, no histopathology available)?
Adherence and compliance to treatment/interventions	Yes/no
Presence of risk factors for vasa previa	List all risk factors (eg, assisted reproductive techniques, twin or higher-order gestation, placenta previa/low-lying placenta, bilobed or succenturiate lobe placenta, velamentous cord insertion)
Antenatal corticosteroid injections	Yes/no plus timing and indication
Care continuity	Yes/no
Antenatal monitoring of fetal growth and well-being	Yes/no, method
Reduced fetal movements during pregnancy	Yes/no
Isolated structural fetal anomalies (diagnosed by ultrasonography or postnatally)	Yes/no, description
Peer support services	Yes/no, description
Pain relief and anesthetic for birth	Description
Patient care satisfaction	Yes/no, specify measure used
Abnormal fetal heart rate pattern in pregnancy and/or labor	Specify abnormality and classification used
Fetal growth restriction	Specify centile and chart used
**Only relevant to studies in which antenatal screening for vasa previa is performed**	
Type of vasa previa	I, II, III, or combination based on ultrasonography findings
Screening for vasa previa by trained professionals at the routine anatomy ultrasonography scan	Yes/no; if no, why (ie, no prescreening mandate)?
Use of published clinical guidelines to diagnose vasa previa (based on distance of exposed fetal vessel to the os at first and last ultrasonography prior to birth, gestational age at which the diagnosis was made, and whether the diagnosis was made using transabdominal or transvaginal ultrasonography)	Yes/no, include name of guideline/criteria
Inpatient vs outpatient management in pregnancy	Include reason (eg, unit protocol, patient preference)
Parental comprehension of diagnosis of vasa previa; its implications and evidence to guide care	Yes/no
Antenatal monitoring for fetal anemia during pregnancy using ultrasonography	Yes/no
Cervical length monitoring using ultrasonography	Yes/no
Change in diagnosis on subsequent ultrasonography (eg, vasa previa not confirmed, vasa previa resolved)	Yes/no; if yes, gestational age at first diagnosis and change in diagnosis

^a^ These items, although not necessarily outcomes, are important to report in studies on vasa previa when possible and relevant. We suggest providing a justification when not reported.

## Discussion

In this survey study involving a 2-round Delphi survey, small group discussions, and a consensus meeting, the COVasP study team obtained consensus from an international group of individuals with lived experience of vasa previa and clinicians and researchers involved in the screening, diagnosis, and management of vasa previa. The final outputs included a 13-item core outcome set of 8 fetal/neonatal and 5 maternal outcomes and a 22-item reporting checklist to be used as applicable. While COVasP represents a minimum set of items to be reported in future vasa previa studies, it should not dissuade researchers from reporting on additional outcomes depending on the focus of specific studies. We hope that COVasP, which represents international consensus between diverse groups of HSUs and HCPs, will find widespread applicability across varied study designs, despite international variations in vasa previa screening, diagnosis, and management.

A systematic review on outcome reporting in vasa previa studies identified inconsistent reporting of patient-important outcomes.^8^ For example, NICU admission was reported in only 19.3% (31 of 160) of studies, while maternal quality of life and neurocognitive and developmental outcomes were not reported in any study. Implementing COVasP may standardize reporting of these essential outcomes endorsed by HSUs and HCPs, ensuring that recommendations in future clinical guidelines are based on outcomes considered important by these groups.

### Strengths and Limitations

The greatest strength of this study is that throughout all stages, international representation and patient interests were maintained, with HSUs representing at least 50% of the participants at each stage. Equal representation of HCPs and HSUs throughout the process ensured that each stage was collaborative and inclusive, with all stakeholder groups’ opinions being considered. International participation allows for its implementation worldwide, as global management and screening protocols for vasa previa were considered at every stage of the process.^20^

The study also has several limitations. First, and most importantly, was participant attrition. The 36% attrition rate observed between rounds is a recognized limitation in Delphi survey studies and is consistent with reported rates even prior to the COVID-19 pandemic.^21,22^ While missing data could introduce bias, attrition rates were comparable between the HCP and HSU groups, and each step still maintained diverse representation across both groups, reducing the likelihood of systematic bias in the final consensus. Nonetheless, the possibility remains that the views of participants lost to follow-up differ from those who completed both rounds.

Second, there are some limitations inherent to the COMET methodology. These limitations include a burden on participants to score a high number of items,^23^ the possibility of no items achieving consensus to be excluded,^24,25^ and disagreement on the effectiveness of a core outcome set with a large number of final outcomes.^24,26^ To address these challenges, and strengthen our approach, we adapted the original protocol, which involved the research team rewording and combining outcomes and introducing small group discussions to vote on newly constructed outcomes before a final consensus meeting.

Third, because vasa previa is a very rare condition, the 2-round Delphi process required participants to rate 67 items. To reduce participant burden, we limited the demographic survey to very few questions, which were optional. While we do not believe this process compromised study rigor, and in fact, encouraged HSUs and HCPs from 27 countries to participate, we were not able to report on participants’ age, sex and gender, and race and ethnicity.

Fourth, the definition of outcome may vary depending on a study’s purpose or subject area.^27^ While some items may serve as outcomes in 1 study, they may function as risk factors in another.^24,27^ Throughout our study, consistent feedback from HSUs and HCPs highlighted that certain items, while not fitting the strict definition of an outcome, were crucial for understanding the management, screening, or presentation of vasa previa. Based on this feedback, a reporting checklist was developed, consisting of items that are essential to capture and report whenever possible but that do not represent outcomes of vasa previa screening or management.

Fifth, the Delphi process, small group discussions, and consensus meeting were administered in English. While this decision optimized feasibility, it may have introduced selection bias favoring participants from predominantly English-speaking, high-income countries. Although the COVasP study team recruited HSUs and HCPs from 27 high-, middle-, and low-income countries, many of which are not primarily English speaking, future iterations of COVasP and other core outcome sets should consider translating material into as wide a range of languages as possible to enhance accessibility and inclusivity, ensure international representation, and minimize language-related biases, thereby strengthening their external validity and global applicability.

Sixth, although the number of final outcomes and reporting checklist items may seem high, this is typical for core outcome sets developed for pregnancy-related conditions, which range from 11 to 40 items.^24,26^ Importantly, we emphasize that COVasP captured aspects of screening and management of vasa previa that affect both mother and infant.

Seventh, we prioritized the inclusion of all patient-important outcomes, while feasibility and resource availability were secondary considerations. Thus, we acknowledge that not all items included in COVasP are feasible to collect for all studies and in all settings. However, researchers should not feel dissuaded from striving to report on these items and clearly justifying the inability to include certain items. We hope that this flexibility will enable researchers to lobby funding organizations and governments to facilitate gathering of data on patient-important outcomes.^28^

Finally, while COVasP has identified core items for studies on vasa previa, a need for consensus on definitions and measurement tools remains. For example, severe neonatal morbidity may be defined using published indicators, such as the Neonatal Adverse Outcome Indicator^29^ or Core Outcomes in Neonatology,^30^ or may be researcher defined. Similarly, several working definitions of severe maternal morbidity exist, including one by the Centers for Disease Control and Prevention^31^ and another by the Canadian Perinatal Surveillance System.^32^ Agreeing on consensus definitions may require collaboration among pregnancy care practitioners and methodologists to ensure consistent reporting practices across studies.^33^ For now, researchers are encouraged to use and reference the most up-to-date definitions endorsed by professional organizations. Schematics defining fetal/neonatal loss, gestational age, and birthweight have recently been published.^34^

We anticipate that the widespread use of COVasP may enhance research quality by ensuring consistent reporting of outcomes and other essential items in diagnostic and management studies on vasa previa, thereby facilitating better data for future meta-analyses and inclusion of outcomes important to both HCPs and HSUs.

## Conclusions

In this survey study involving Delphi surveys, small group discussions, and a consensus meeting, collaboration among an international group of HSUs and HCPs resulted in the establishment of a core outcome set and reporting checklist for future studies on vasa previa. These tools are available in fillable format aimed at standardizing outcome selection, incorporating patient-important outcomes into research, harmonizing data synthesis, and facilitating the development of patient-centered clinical guidelines for this rare, but potentially devastating condition.

## Notes

Supplement 1. **eAppendix.** A Core Outcome Set and Reporting Checklist for Studies on Vasa Previa

Supplement 2.
Data Sharing Statement
