# Supplementary material for: Core Outcome Set and Reporting Checklist for Studies on Vasa Previa
Source: JAMA Netw Open. 2025 Mar 18;8(3):e251000. doi: 10.1001/jamanetworkopen.2025.1000 (PMC11920838; doi:10.1001/jamanetworkopen.2025.1000)
Supplement: Supplement 1. — eAppendix. A Core Outcome Set and Reporting Checklist for Studies on Vasa Previa [file jamanetwopen-e251000-s001.pdf]

Supplemental Online Content

Yeretsian T, Javid N, Hirschhorn-Edwards N, et al. Core outcome set and reporting checklist for studies on vasa previa. *JAMA Netw Open*. 2025;8(3):e251000. doi:10.1001/jamanetworkopen.2025.1000

eAppendix. A Core Outcome Set and Reporting Checklist for Studies on Vasa Previa

This supplemental material has been provided by the authors to give readers additional information about their work.

© 2025 Yeretsian T et al. *JAMA Network Open*.

eAppendix. A Core Outcome Set and Reporting Checklist for Studies on Vasa Previa

A fillable version of the Core Outcome Set and Reporting Checklist for Studies on Vasa Previa is available at https://www.clarityresearch.ca/core-outcome-set-and-reporting-checklist-for-studies-on-vasa-previa

© 2025 Yeretsian T et al. *JAMA Network Open*.
