# Supplementary material for: Core Outcome Set and Reporting Checklist for Studies on Vasa Previa
Source: JAMA Netw Open. 2025 Mar 18;8(3):e251000. doi: 10.1001/jamanetworkopen.2025.1000 (PMC11920838; doi:10.1001/jamanetworkopen.2025.1000)
Supplement: Supplement 2. — Data Sharing Statement [file jamanetwopen-e251000-s002.pdf]

Data Sharing Statement

Yeretsian. Core Outcome Set and Reporting Checklist for Studies on Vasa Previa. *JAMA Netw Open*. Published March 18, 2025. doi:10.1001/jamanetworkopen.2025.1000

Data
Data available: No
